# Soil fertility impact on recruitment and diversity of the soil microbiome in sub-humid tropical pastures in Northeastern Brazil

**DOI:** 10.1038/s41598-024-54221-7

**Published:** 2024-02-16

**Authors:** Diogo Paes da Costa, Thallyta das Graças Espíndola da Silva, Ademir Sérgio Ferreira Araujo, Arthur Prudêncio de Araujo Pereira, Lucas William Mendes, Wisraiane dos Santos Borges, Rafaela Felix da França, Carlos Alberto Fragoso de Souza, Bruno Alves da Silva, Renata Oliveira Silva, Erika Valente de Medeiros

**Affiliations:** 1 Microbiology and Enzimology Lab., Federal University of Agreste Pernambuco, Garanhuns, PE 55292-270 Brazil; 2 Soil Quality Lab., Agricultural Science Center, Federal University of Piauí, Teresina, PI 64049-550 Brazil; 3 Microbial Ecology and Biotechnology Lab., Federal University of Ceará, Fortaleza, CE 60020-181 Brazil; 4 Center for Nuclear Energy in Agriculture, University of Sao Paulo, Piracicaba, SP 13400-970 Brazil

**Keywords:** Soil microbiology, Environmental impact, Microbial ecology

## Abstract

Soil fertility is key point to pastures systems and drives the microbial communities and their functionality. Therefore, an understanding of the interaction between soil fertility and microbial communities can increase our ability to manage pasturelands and maintain their soil functioning and productivity. This study probed the influence of soil fertility on microbial communities in tropical pastures in Brazil. Soil samples, gathered from the top 20 cm of twelve distinct areas with diverse fertility levels, were analyzed via 16S rRNA sequencing. The soils were subsequently classified into two categories, namely high fertility (HF) and low fertility (LF), using the K-Means clustering. The random forest analysis revealed that high fertility (HF) soils had more bacterial diversity, predominantly Proteobacteria, Nitrospira, Chloroflexi, and Bacteroidetes, while Acidobacteria increased in low fertility (LF) soils. High fertility (HF) soils exhibited more complex network interactions and an enrichment of nitrogen-cycling bacterial groups. Additionally, functional annotation based on 16S rRNA varied between clusters. Microbial groups in HF soil demonstrated enhanced functions such as nitrate reduction, aerobic ammonia oxidation, and aromatic compound degradation. In contrast, in the LF soil, the predominant processes were ureolysis, cellulolysis, methanol oxidation, and methanotrophy. Our findings expand our knowledge about how soil fertility drives bacterial communities in pastures.

## Introduction

In Brazil, pastures cover ~ 154 million hectares where about 65% exhibit signs of intermediate to severe degradation^1^. The pasture degradation reduces its capacity to produce biomass to support animals and maintain the ecosystem productivity. In tropical soils, the process of degradation is associated to overgrazing and reduced soil fertility^2^. Consequently, when converting native forests into pastures, it becomes necessary to enhance soil conditions. This involves increasing soil pH and exchangeable bases, while simultaneously reducing aluminum content and potential acidity^3^. Conversely, the transformation of native forests into pastures leads to a reduction in soil organic carbon content, a factor that contributes to degradation process^4,5^. Furthermore, the consequences of overgrazing—where pastures are burdened with more animals than they can sustain—are far-reaching. This not only drastically diminishes soil cover but also accelerates the desertification process^2^. The ripple effect of this is a significant reduction in the reservoirs of Carbon and Nitrogen in the soil, which has a profound impact on bacterial communities, disrupting the delicate balance of our ecosystem^3–5^. Furthermore, improved soil management, such as that promoted by integrated agricultural systems, can provide additional benefits. These include animal welfare and adaptation, as well as mitigation of climate change^6^.

The bacterial communities are essential to nutrient cycling in the soil, and consequently to proper ecosystem functioning^6^. While numerous publications have documented the soil bacterial communities in subhumid tropical pastures^3–5,7,8^, the relationship between soil fertility and the soil bacteriome in these pastures remains not fully understood. This is an area that continues to be explored in the scientific community. Indeed, previous studies have mostly focused on specific driver, such as specific nutrients and soil organic carbon^4,5,7–9^. For instance, Bastida et al.^8^ have reported that soil microbial diversity and biomass ratios are highest in arid environments with low carbon content, while Costa et al.^7^ revealed strong association between the quality of pastures in a sub-humid tropical region and the participation of organic carbon fractions and microbial biomass. These studies also reported that proper pasture management practices can significantly enhance microbial diversity and complexity, with pastures exhibiting no distinction from adjacent preserved forests.

Although knowledge about the soil microbiome in tropical regions has advanced in recent years^3,4,6–8^, especially about the occurrence of predominant taxonomic groups^3,8^, the knowledge on bacterial composition, structure and ecological interactions with soil fertility are still in diffusion^7^. These gaps need to be elucidated, including the interactions that modulate the bacteriomes of soils under pasture environments and their fertility^9^. Moreover, further studies focused on changes in microbial structure and their correlation with enzymatic processes are necessary for the development of new indicators of ecosystem health and sustainability^10^. Given the importance of functional predictions for guiding management practices that promote soil health and productivity, as well as the enrichment of beneficial microbial communities, it is crucial to understand the highlighted factors. In this study, we hypothesized that different levels of soil fertility, as classified by K-means clustering (Table S1), would influence the composition and function of microbial communities. Thus, the aim of this study was to assess the relationship between soil fertility levels in pasture fields and the characteristics of bacterial communities, including their structure, diversity, composition, co-occurrence, and functionality, using advanced statistical methods.

## Results

### Identification of soil fertility clusters by K-means

**Figure 1:**
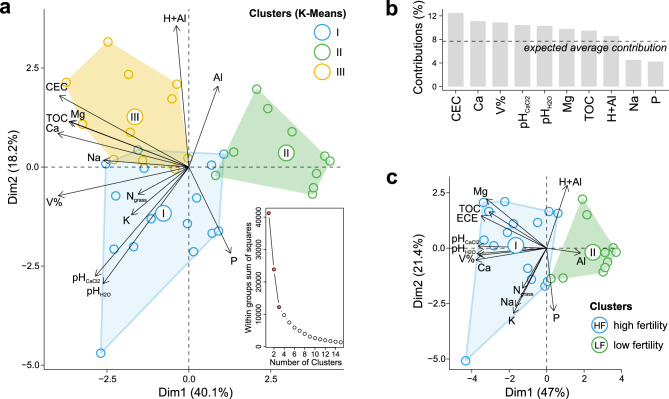
Grouping of pastures through principal components and K-Means clustering algorithm based on soil chemical attributes and leaf nitrogen content. (**a**) Biplot of the principal component analysis (PCA) highlighting the three most important clusters according to the K-Means grouping; (**b**) Contribution of the main soil variables to the variance explained by the two main axes of the PCA; (**c**) New PCA biplot highlighting the difference in soil fertility levels between the two most contrasting clusters. *CEC* cation exchange capacity, *N*_*grass*_ leaf-N, *TOC* total organic carbon, *V%* base saturation. Created in the R environment (v.4.3.1).

The analysis of the total sum of squares within groups (SSW), classifying soils by the K-Means algorithm, showed a sharp drop in variability occurring from the division of samples into three clusters (k = 3, between_SS/total_SS = 42.2%) (Fig. 1a). These results demonstrate that by assigning samples to ‘k’ clusters instead of ‘n’ (number of samples), the clusters achieved a 42.2% reduction in explaining total variability, and little was added with the gradual increase in the number of k-groupings, suggesting a comparative study of the three groups or between the two most contrasting. The principal component analysis (PCA) explained 58.3% of the total environmental variability. Both clusters I and II, located in opposite quadrants, presented contrasting associations for most soil variables, mainly pH, and available Al, and leaf-N. Overall, greater variations of the multivariate model were attributed to CEC, followed by Ca^+^, V%, pH_CaCl2_, pH_H2O_, Mg^2+^, TOC, and H + Al (Fig. 1b).

The K-means analysis subdivided the samples into groups of 15 (I), 10 (II), and 11 (III). Groups I and II were used in the downstream analyses, corresponding to the soils with the most significant contrasts among the studied variables, primarily pH and cation saturation (Table S1). The isolation of clusters I and II increased the explanatory power (68.4% of variability) of PCA, where there was a clear distinction in soil fertility (Fig. 1c). Most environmental variables, favorable to soil fertility, showed significant (p < 0.05) and positive correlations with the first dimension of PCA (pH, CEC, TOC, and all the cations from the saturation calculation) and the opposite was observed for Al^3+^ and H + Al (Table S3). In this case, cluster-I continued to be strongly associated with chemical attributes that positively influenced soil fertility (high fertility—HF) while cluster-II was characterized by more acidic soils, poor (lower V% and CEC—total cation exchange capacity) and with significant predominance of Al^3+^ (low fertility—LF) (Fig. 1c). These results suggested that the comparative analysis of LF and HF clusters demonstrated the greatest probability of identifying possible impacts of changes in pasture soil fertility on the parameters of structure, diversity, composition, and interaction of microbial communities.

Statistical tests confirmed significant superiority of the means of pH, Ca^2+^, Mg^2+^, TOC, CEC, and V% in HF soil compared to LF soil (Table S1, Fig. S3). The opposite was observed for Al^3+^ concentrations (twice the average in HF). The greatest contrast was in base saturation, where HF soil had an average of 68.1 ± 8.1% while in LF soil the V% was only 31.4 ± 19.8%.

### Associations between soil attributes and bacterial diversity

**Figure 2:**
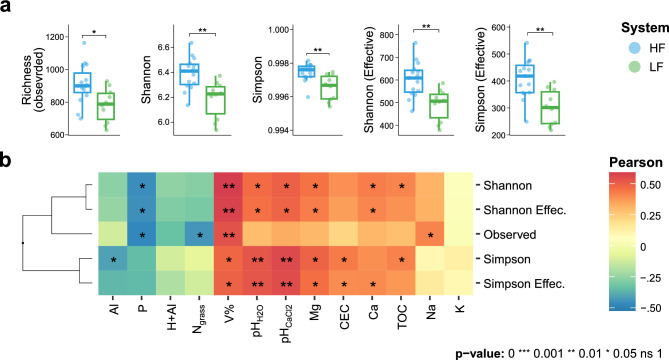
Alpha-diversity metrics and their associations with the chemical attributes of HF and HL pasture soils. (**a**) Comparisons of diversity indices by Wilcoxon signed-rank statistics; (**b**) Monotonic associations between alpha-diversity and chemical variables through Pearson correlation coefficients. Associations marked with one asterisk (*) or more were considered significant. *CEC* cation exchange capacity, *Effec.* Effective number of ASVs based on the Shannon and Simpson indices, *N*_*grass*_ leaf nitrogen in the aerial part of the pastures, *TOC* total organic carbon, *V%* base saturation in the soil. Created in the R environment (v.4.3.1).

All alpha-diversity indices were higher in HF soil (Wilcoxon, p-value < 0.05) and the Shannon and Simpson indices showed similar results to their respective diversity values recalculated as ASVs effective numbers (Fig. 2a, Table S4). Considering the effective diversity, HF soil had on average about 111 and 107 more ASVs than LF soil, based on the Shannon and Simpson indices, respectively (Table S4).

Bases saturation (V%) was the most prominent variable in terms of positive contribution to alpha-diversity indices, followed by pH and Mg^2+^ concentration (Fig. 2b). Although the Shannon and Simpson diversity indices were positively affected by TOC, their respective effective numbers were not. Additionally, the calculation of the effective number of ASVs, through Simpson, showed a strongly positive correlation with Ca^2+^, unlike the Simpson index, revealing significant contrasts between both calculations. Cation exchange capacity (CEC) demonstrated significant positive influence on both indices based on Simpson. In contrast, the concentrations of labile-P, exchangeable Al, H + AL and leaf-N showed negative correlations with all observed indices. In this case, P stood out, showing significant negative influence on the total number of ASVs (richness) and on Shannon-based indices. In addition, richness and Simpson’s diversity were significantly reduced by the contents of leaf-N and exchangeable Al, respectively.

**Figure 3:**
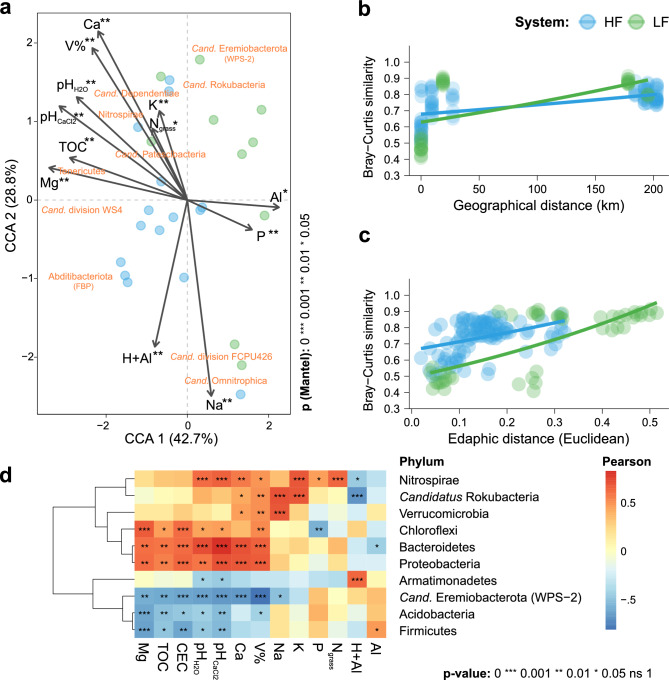
Beta-Diversity analysis of microbial communities in pastures with high (HF) and low fertility (LF) soils. Biplot with canonical correlation analysis (CCA) based on generalized UniFrac distance highlighting significant environmental variables (**a**), according to the Mantel test (p < 0.05), and the main responsive phyla. The average similarity between samples, calculated by Bray–Curtis dissimilarity, was associated with geographic distances (**b**) and edaphic distances (**c**). The phyla with significant correlations with one or more variables were also analyzed (**d**). *CEC* cation exchange capacity, *N*_*grass*_ leaf-N, *TOC* total organic carbon, *V%* base saturation. Created in the R environment (v.4.3.1).

According to the canonical correlation analysis (CCA) using generalized UniFraq distance (Fig. 3a), the structure of microbial communities showed a segregation pattern like that observed in the PCA of chemical attributes (Fig. 1c), explaining an even greater proportion of the total variation (70.5%). There was a strong positive participation of pH, Ca^2+^, Mg^2+^, V%, and TOC in HF soil, highlighting the abundance of the bacterial phyla *Candidatus* Dependetiae, Nitrospirae, *Candidatus* Patescibacteria, and Terericutes in this niche, as well as *Candidatus* division WS4 and Abditibacteriota (old *Candidatus* FBP). In this model, the positive association of available P and Al^3+^ levels in LF soil also became clearer, where the *Candidatus* Eremiobacterota (old *Candidatus* WPS-2) phylum stood out and *Candidatus* Rokubacteria less intensely. Considering that some samples that made up each cluster were collected between distances of up to 200 km (Fig. 3b), this factor did not demonstrate variability in beta-diversity (Distance-Decay) greater than that generated by soil fertility patterns (Fig. 3c), resulting from the K-Means clustering.

### Taxonomic composition and enrichment

Six phyla were positively correlated with several beneficial chemical attributes of the soil, mainly pH (in H_2_O and CaCl_2_), V%, K^+^, Ca^2+^, Mg^2+^, CEC, and TOC (Fig. 3d). Prioritizing according to the number of positive and significant associations, the Bacteroidetes and Proteobacteria phyla stood out, that showed highly significant abundance with pH, V%, Ca^2+^, Mg^2+^, CEC, and TOC content. In the third position, the phylum Chloroflexi stood out, correlating positively and significantly with all these variables, except for Ca^2+^. Lastly, the phyla Nitrospirae, Verrucomicrobia, and *Candidatus* Rokubacteria stood out, all with positive abundance and significantly associated with V%, primarily driven by Ca^2+^. Nitrospira and *Candidatus* Rokubacteria were the only ones to show positive and significant correlations with K^+^, only Nitrospira demonstrated the same for P and N_grass_ (pasture leaf N). Others were predominantly associated with less fertile soils, highlighting the *Candidatus* Eremiobacterota (WPS-2), followed by the Acidobacteria and Firmicutes phyla, negatively and significantly associated with pH, ECE, TOC and Mg^2+^ contents.

**Figure 4:**
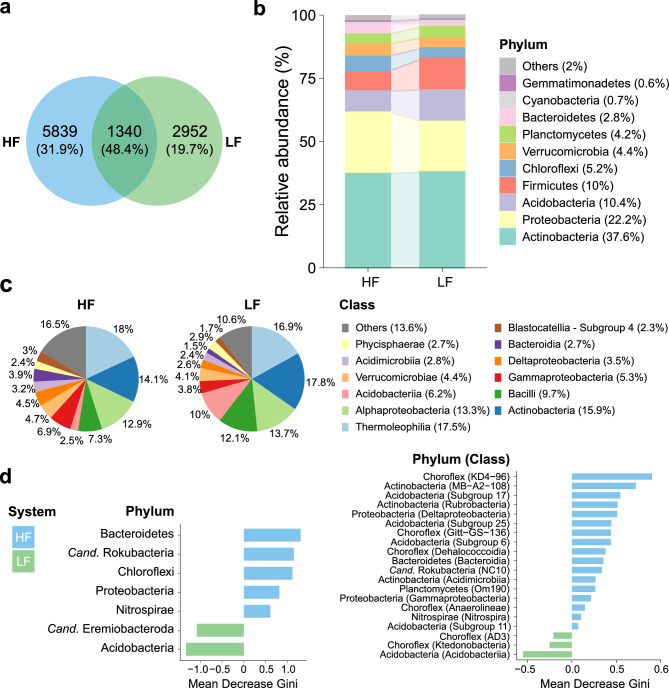
Relative composition and differential abundance of the main bacterial taxonomic ranks found in rich (HF) and poor (LF) pastures in fertilization. (**a**) Venn diagram showing the percentage of ASVs unique to each niche and shared between both; (**b**) relative abundance of the ten most abundant bacterial phyla; (**c**) relative abundance of the 12 most abundant classes; (d) Differential abundance analysis based on the taxon importance estimator (phyla and classes) in the decision tree branched by the Random-Forest algorithm (Mean Decrease Gini). Created in the R environment (v.4.3.1).

The abundance analyses identified differences between HF and LF soils in terms of enrichment of the main taxonomic ranks (Fig. 4). Initially, it was observed that 48.4% of the ASVs were shared between both clusters (Fig. 4a). However, HF soil presented the largest set of unique ASVs (31.9%) compared to LF soil (19.7%). Overall, the Actinobacteria phylum was the most abundant, representing 37.6% of all sequences (Fig. 4b). Next were the Proteobacteria (22.2%), Acidobacteria (10.4%), Firmicutes (10%), Chloroflexi (5.2%) and the others did not exceed 5% relative abundance. At the general class level, Thermoleophilia (17.5%), Actinobacteria (15.9%), Alphaproteobacteria (13.3%), Bacilli (9.7%), Acidobacteria (6.2%) and Gammaproteobacteria (5.3%) and Verrucomicrobiae (4.4%) predominated, with the others not exceeding 4% relative abundance (Fig. 4c). Comparing the two clusters, the Acidobacterria class showed the greatest variation, with a relative proportion about four times higher in LF.

The differential abundance analysis through Random-Forest confirmed that HF soil was significantly enriched by Bacteroides, Rokubacteria, Chloroflexi, Proteobacteria, and Nitrospirae (Fig. 4d), while the Acidobacteria and *Candidatus* Eremiobacterota were more represented in LF soil. Most of the significantly enriched classes also concentrated in HF soil, highlighting Gammaproteobacteria, Deltaproteobacteria, Acidimicrobiia, and Bacteroidia for being among the 12 most abundant classes. Another thirteen classes with a relative abundance of less than 1% (each) were also enriched in HF soil. Acidobacteriia was the class highlighted in LF soil, where two less common groups also emerged, Ktedonobacteria class and uncultured Chloroflexi (AD3) sequences.

### Species co-occurrence in ecological interaction networks

**Figure 5:**
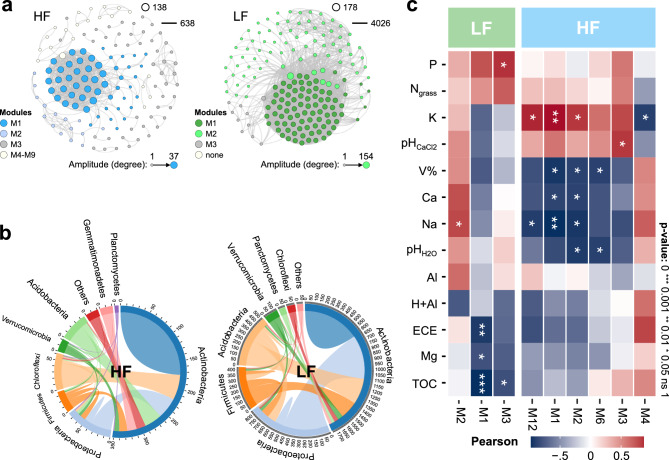
Microbial co-occurrence analyses in pastures on fertile (HF) and poor (LF) soil highlighting highly correlated groups (ASVs) through the SPIEC-EASI association measure (SparCC > 0.6, p < 0.01). (**a**) Networks, where modules were differentiated by colors and the degrees (number of connections) were directly proportional to the node diameter; (**b**) Abundance of connections at the bacterial phylum level. Values around circles represented the number of edges connected between phyla or within the same phylum. (**c**) Autogenic analysis of modules that showed significant association with at least one environmental variable. *CEC* cation exchange capacity, *N*_*grass*_ leaf-N, *TOC* total organic carbon, *V%* base saturation. Created in the R environment (v.4.3.1).

Although HF soil stood out significantly in terms of most richness and diversity parameters, the co-occurrence study identified that in LF soil there was greater complexity of significant interactions (C = 22.6) (SparCC > 0.06, p < 0.01) than in HF soil (C = 4.6), being a measure established by the ratio between the number of edges and nodes of ASVs (C = edges/nodes) (Fig. 5a). The range of interaction degrees (number of edges at each node) mirrored this result, where the maximum connection established by an ASV in HF soil was 37 edges, while in LF soil it reached up to 154. The average degree of the network in LF soil (46.54) was also higher than in HF soil (9.25), indicating a predominance of a few modules characterized by nodes with a higher number of connections (M1, M2, and M3). In the other cluster, HF soil presented, in addition to these main modules, numerous other ecological sub-systems operating in the network. The centralization and density of the network in LF soil were also higher (Table S5). Despite the lower complexity of HF soil, this network presented the highest number of positive connections, 66% versus 58% of LF soil, suggesting a system where the occurrence of most bacteria happens in an integrative manner. In addition, the network in HF soil presented a higher diameter, average path length, heterogeneity, and, more slightly, clustering coefficient (Table S5). Overall, the clustering coefficient values (> 0.4) for HF and LH soils suggest a high propensity for module formation in the network (Table S5).

Regarding the taxonomic composition of the networks (Fig. 5b), most of the connections in both systems were established by ASVs assigned to the most abundant phyla, being Actinobacteria (HF ~ 350; LF ~ 1800) and Proteobacteria (HF ~ 80; LF ~ 850). From these taxa, there was greater differentiation between the compositions of the networks. In this sense, a higher relative number of Choroflexi connections was observed in HF soil (~ 60) and Acidobacteria in LF soil (~ 500). The Gemmatimonadetes phylum only appeared in the HF network (~ 20 interactions), being the tenth most abundant phylum overall (Fig. 4b).

The autogenic analysis of the network modules, equivalent to the correlation between the first principal component (PCA) of each module with the environmental data, showed significant effects of the chemical attributes of the soils on the main modules of each network (Fig. 5c). The HF system presented a greater number of significantly affected components, highlighting the negative effect of V% and Ca^2+^ and Na^+^ concentrations on the main and most abundant modules (M1 and M2). On the other hand, soil K availability seems to favor ASVs that integrate these modules in HF soil. In LF soil, module M1 correlated negatively with cation exchange capacity (CEC) and with Mg^2+^ and total organic carbon (TOC) concentrations, while M2 was positive for Na^+^. TOC also inhibited module M3, while available P content stimulated it. In both M1 and M2 modules, Actinobacteria dominated (avg. 54.6%) the taxonomic composition (Table S6). Proteobacteria was the next most common (avg. 18.2%), except in LF soil M2 (12.7%), where Firmicutes (24%) and Acidobacteria (16.5%) took precedence. In summary, these phyla are keystones in biological interactions in nutrient-poor pasture soils, along with others that were less participatory in the network (Choroflexi, Planctomycetes, and Verrucomicrobia). Together, these three auxiliary phyla in the network represented an average of 7% of the ASVs components of modules M1 and M2 (Table S6).

### Functional prediction

**Figure 6:**
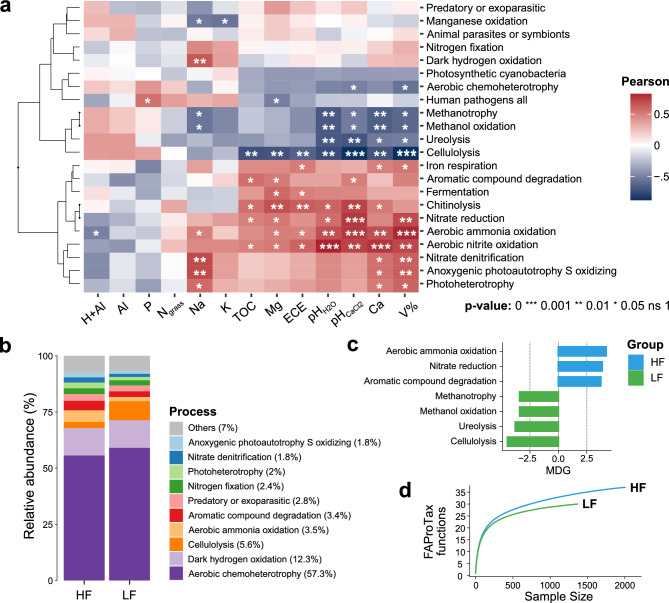
Metagenomic prediction analysis based on the abundance of 16S rRNA genes associated with functional profiles from the FAProTax database. (**a**) correlations between environmental variables and predicted functional profiles; (**b**) relative frequency of the most abundant functional processes among clusters; (**c**) differential abundance of processes significantly distinct between HF and LF according to Mean Decrease Gini (MDG); (**d**) Richness of predicted functions depending on sample size. *CEC* cation exchange capacity, *N*_*grass*_ leaf-N, *TOC* total organic carbon, *V%* base saturation. Created in the R environment (v.4.3.1).

The results of the functional prediction showed more associations between predicted processes and the most favorable edaphic parameters for soil fertility (Fig. 6a). V% stood out, positively associated with pathways related to the nitrogen cycle, highlighting aerobic ammonia oxidation followed by aerobic nitrite oxidation, nitrate reduction, and nitrate denitrification, as well as iron respiration, anoxic photoautotrophy, and oxidizing photoheterotrophy. Similar associations were observed for the variables pH (general), Ca^2+^, Mg^2+^, and CEC. In general, a greater number of ASVs were associated with the processes of aerobic chemoheterotrophy (57.3%), followed by dark hydrogen oxidation (12.3%), cellulolysis (5.6%), and aerobic ammonia oxidation (3.5%) (Fig. 6b). Among all these processes, linear discriminant analysis (LDA) allowed to distinguish the enrichment of seven ASVs between clusters (Fig. 6c). In HF soil, a significant number of ASVs were attributed to the processes of aerobic ammonia oxidation, nitrate reduction, and degradation of aromatic compounds. In LF soil, associations with cellulolysis, ureolysis, methanotrophy, and methanol oxidation stood out. In conclusion, the results showed that in LH soil there was a greater enrichment of ASVs associated with FAProTax functions compared to LF soil, inferring that soil fertilization favored the richness of bacterial genes related to soil element cycling (Fig. 6d).

In addition to these results, the molecular functions predicted in this study, based on the annotation of the 16S rRNA gene, showed a high degree of association with the annotation based on Shotgun metagenomic sequencing using the eggNOG database (ρ = 0.93, p < 0.001). In this case, we can conclude that, under the strict conditions of this study, it was possible to infer functional profiles with good accuracy using data from the V3-V4 region of the 16S (Fig. S4).

## Discussion

This study provides information on how soil fertility affected the structure of microbial communities in pastures in a sub-humid tropical zone from Northeast, Brazil. In addition, our results provided valuable information for researchers studying microbial communities in pastures and how they are affected by soil fertility. The use of both supervised (Random Forest) and unsupervised (K-Means) machine learning methods was relevant to identify patterns and differentiating keystone bacterial species in response to distinct soil fertility levels. The findings suggest that fertile soils exhibited a higher diversity (Fig. 2a) and predominance of important bacterial phyla, mainly Proteobacteria, Nitrospira, Chloroflexi, and Bacteroidetes, while poor soils favored Acidobacteria (Fig. 4d). Moreover, fertile soils showed fewer significant interactions (Fig. 5a), but a greater number of independent interactive modules, where correlations between microorganisms were predominantly positive (Table S5). Previous studies have reported that increased soil fertility positively affected the diversity^3,7,8^, functions^9,10^, and modularization of microbial co-occurrence networks^9^, attributing a more relevant role to bacteria in maintaining soil fertility and crop productivity^11^. However, our study showed that bacterial communities in different soils respond similarly to fertility levels, as emphasized by the K-means method, driving microbial community recruitment, irrespective of the sample geographical location.

The use of statistical methods, such as K-means and Random Forest algorithms, was crucial in assessing the responses of microbial communities to soil properties in this study. These approaches, as highlighted by Havatu et al.^12^, Ghannam and Techtmann^13^, have proven to be effective tools in understanding the complex interactions within microbial communities and their environment. The K-Means consists of an unsupervised method that can be applied to identify clusters of regions based on their soil characteristic^12^, being considered an efficient method for comprehensive evaluation of soil fertility^14^. This tool can be associated with the use of the supervised Random Forest method to make predictions or classifications based on labeled environmental genomic DNA data^13^. Interestingly, the Random Forest tool is considered one of the most effective machine learning models for analyzing soil microbiome data, demonstrating high classification accuracy with a variety of 16S rRNA data sets^15^.

The results revealed a prevalence of the Nitrospira, *Candidatus* Dependentiae, and *Candidatus* Patescibacteria in more fertile soils, showing a positive association of these phyla with pH, Ca^2+^, Mg^2+^, V%, and TOC according to CCA (Fig. 3a). In addition, the random forest-based classifier confirmed that Nitrospira was significantly higher in fertile soils (Fig. 4d). These results pointed to a sensitive response of Nitrospira to fertilization, characterized by being highly responsive to soil nitrogen concentrations and including bacteria that can catalyze the oxidation of ammonia and nitrite^16^. This was a strong indication that pasture quality was mainly related to bacterial species involved in soil N cycling, contributing to the development of an indicator of pasture soil productivity and health.

Other studies have already highlighted the impacts of fertilization on the taxonomic and functional aspects of nitrogen-cycling microorganisms in pastures. Yang et al.^17^ showed that long-term management of pastures using chicken bedding and cattle manure increased microbial community richness, corresponding to higher soil pH and nutrients, which is consistent with the results of our study (Fig. 2). Similarly, Wakelin et al.^18^ found that agricultural management and fertilization practices had a significant impact on the microbiota of pasture soils, altering the quantification of genes involved in nitrification (*amo*A), denitrification (*nar*G), and nitrogen fixation (*nif*H). Liu et al.^11^ also demonstrated that both soil fertility and management influenced the structure of edaphic communities in semi-arid fields in Mongolia, with ammonia-oxidizing bacteria (AOB) and ammonia-oxidizing archaea (AOA) responding positively to urea application in pastures. Together with our present study, these works highlighted the importance of cultivation-independent techniques to access microbial communities important for nutrient cycling in soil. This is justified because most microorganisms cannot be cultivated under laboratory conditions^19^, as is the case for species of the Nitrospira phylum, slow-growing and fastidious Nitrite-Oxidizing Bacteria (NOB)^20^. This information is indicative that soil management is fundamental for the establishment of microbial communities that promote the development of pastures.

While numerous studies have shown the impact of management and soil fertility changes on key ecological processes, particularly nitrogen-related ones^9,11,16–18,21^, they have not emphasized the enrichment or roles of specific microbial taxa across various scenarios. In contrast, our study highlights this for Nitrospira, Patescibacteria, Bacteroidetes, Chloroflexi, Proteobacteria, *Candidatus* Dependetiae, *Candidatus* Rokubacteria, among others (Fig. 4). These last four phyla, enriched in HF soil, also have recognized ecosystem participation in bulk soil and the rhizosphere recruitment process in general^22,23^, in addition to the Actinobacteria (similar occurrence in HF and LF soils) and Acidobacteria (enriched in LF soils) phyla (Fig. 6d).

We applied co-occurrence network analysis to assess the interaction and dynamics of microbial communities. Network analysis is a valuable tool for comprehending the intricate relationships between microbes and their environment^24^. Our findings revealed that HF soil exhibited lower complexity but a higher number of positive connections. Ku et al.^25^ demonstrated that soils with higher pH had a greater proportion of positive connections in co-occurrence networks involving functional N-genes, indicating that increased pH and soil fertility fostered connections between these genes. Similarly, our study observed a prevalence of positive connections and greater modularity in the HF microbial network (Fig. 5a and Table S5). These findings indicate the presence of microbial niches with species engaging in more prevalent and significant interactions, leading to a highly interconnected network^23^.

The number of interactions per unit of nodes (ASVs) observed in the LF network (Fig. 5a), indicates some level of interactive complexity. However, modularity is one of the main components of microbial networks and can indicate a more complex topological structure^26^. Additionally, the complexity of the co-occurrence network may be proportional to the connection between microbial diversity and pasture production^9^. Therefore, this suggests that the increase in nutrient availability in the soil can create conditions like those observed in the rhizosphere zone, where N and TOC are predominant^27^. This naturally favors the growth of copiotrophic microorganisms (r-strategists)^22^. While Acidobacteria, Chloroflexi, Gemmatimonadetes, and Nitrospira are enriched in bulk soil, showing oligotrophic behavior (k-strategists), the Bacteroidetes, Proteobacteria, and Actinobacteria phyla tend to be more recruited by rhizospheres or environments with more resources as they are predominantly copiotrophic groups (r-strategists)^22,23^. In addition, studies indicate that proper maintenance of soil fertility relieves competition for substrates, favoring mutualistic microbes, increasing complexity at the expense of the stability of the co-occurrence network, mainly regarding nitrogen^9,21^. Importantly, the pastures in our study were initially selected based on the reflective vegetation index (NDVI), which significantly reflected the contrast of leaf nitrogen content between HF and LF soils (Fig. S2).

More fertile soils, in parallel with the rhizosphere zone, may recruit fewer species for executive functions, but allocate more modules for swift recycling of key elements, primarily phosphorus, nitrogen, and carbon^27,28^. The smaller number of nodes observed in our HF network reflects the base saturation and showed strong positive correlations with the two main modules of this network, predominantly formed by Actinobacteria and Proteobacteria (Table S6). The maintenance of Actinobacteria communities is extremely relevant for the soil, as this phylum has species with great metabolic versatility, capable of metabolizing chitin, lignin, higher fatty acids, steroids, and humic acids of difficult decomposition^29^. Actinobacteria can also show great resilience to water stress and alkaline pH, in addition to acting in the release of inorganic-N originating from recalcitrant organic matter^30^, making the enrichment of this group in the soil also an interesting fact to promote the development of pastures. It is also worth mentioning the enrichment of the Acidobacteria phylum in LF soil, a group whose genetic sequences represent a significant fraction of the soil microbial community^31^. In addition, Acidobacteria is considered an underrepresented bacterial phylum in soil, with its members being ubiquitous and widely distributed in almost all ecosystems^22^.

The results of FAProTax, a database to predict putative potential functions based on the 16S rRNA gene, showed significant enrichment of the aerobic ammonia oxidation process in HF soil, which is probably associated with the enrichment of species from the Nitrospira phylum (Figs. 4d and 6). This group of aerobic bacteria (chemolithoautotrophic) has species capable of producing nitrate from the oxidation of nitrite, originating from the aerobic ammonia oxidation process, a cation released by other bacterial or archaeal groups^32^, underscores the importance of building microbial co-occurrence networks. This insight underscores the significance of identifying bacteria associated with nitrogen cycling in pasture soils, which can contribute to their sustainability. Luo et al.^33^, which assigned a secondary role to nitrogen input in soil for crop production, implying that nitrogen aids in enhancing the resistance and resilience of functional genes involved in the cycling of nitrogen, carbon, and phosphorus.

Our data also showed that methanotrophy was more abundant in the LF soil (Fig. 6c), which indicates a potential for methane uptake. In a recent study, Souza et al.^34^ showed that, although grass coverage increases methane uptake in pasture soils, liming to increase pH compromised the capacity of the soil to be a sink for methane. Our results reinforce the importance of proper soil fertilization to promote healthy grass coverage that helps mitigate methane emissions in pasture soils. Despite these results, we suggest caution in interpreting the results obtained by the FAProTax tool, considering that its performance can be improved as the taxonomic and functional reference databases are updated^10,58^.

Therefore, the soil microbial composition can serve as an indicator of its biological quality, as it quickly reflects the effects of modifications in pastures^35^, portraying changes in metabolic functions in the soil in general^36^. The loss of microorganisms in these interactive complexes can have severe impacts on nutrient cycling in the soil since high microbial diversity is crucial for proper ecosystem functioning^6^. Our results also showed that soil fertilization contributed to the detection of a greater number of bacteria associated with important functions, corroborating these studies (Fig. 6d). Our study emphasizes the potential advantages of soil fertility management, which may affect microbial diversity. However, it is crucial to understand that only an increase in microbial diversity does not necessarily enhance soil functioning due to the interconnectedness of soil bacterial structure and fertility^10^, although this is essential for proper ecosystem functioning^6,25^. Consequently, any positive effect on overall rangeland health is likely a result of improved soil properties rather than solely increased microbial diversity^3,6–9^.

This study underscores the significance of prioritizing more abundant Amplicon Sequence Variant (ASV) in microbial community research. These ASVs, backed by multiple sequences, offer solid and trustworthy analyses of the diversity, composition, relative abundance, correlation, and co-occurrence, representing the dominant members of the microbial community^6,7,22,23,29,58^. Thus, their abundance patterns are likely to be more biologically meaningful^23,29^. This approach enhances the validity and interpretability of the study results. Therefore, while all ASVs contribute to microbial diversity, the more abundant ones offer a more accurate understanding of the community structure and function.

Our research successfully utilized K-Means clustering to accurately classify soils into high (HF) and low fertility (LF) levels of pasture in the subhumid tropical of Northeastern Brazil (Fig. 1). This allowed us to identify significant differences in various parameters related to microbial communities, including structure, diversity, composition, connectivity, and functionality. We found that HF soil had higher alpha-diversity parameters, with a predominance of important bacterial phyla such as Proteobacteria, Nitrospira, Chloroflexi, and Bacteroidetes, as well as rarer phyla such as Nitrospirae and Rokubacteria (Figs. 2 and 4). In contrast, LF soil favored Acidobacteria due to their lower pH (Fig. 3). Furthermore, HF soil had fewer microbial connections and were composed of a larger number of modules (Fig. 5), indicating the presence of multiple interaction nuclei within a larger set. Moreover, functional prediction revealed that, in HF soil, bacterial species related to N-cycling were more enriched in HF soil (Fig. 6), while cellulolytic activity and methanotrophy were more abundant in LF soil. Despite the difference in relative abundances calculated by the two methods, it is important to highlight that our FAProtax-based metagenomic prediction demonstrated a high non-linear (Spearman) correlation with the annotated functions from Shotgun sequencing. This indicates a strong association in the frequency of occurrence of annotated genes.

Understanding the interaction between soil fertility and microbial communities is crucial for effective pasture management. Properly adjusting pH, Ca^2+^, Mg^2+^, V%, and TOC levels can promote microbial diversity, essential for proper ecosystem functioning^6,25^, and favor bacterial species involved in the nitrogen cycle, such as Nitrospira, contributing to pasture soil productivity and health^16^ (Figs. 4d and 6). The use of statistical methods like K-Means and Random Forest can aid in monitoring and predicting microbial community responses to soil properties^12,13^. While an increase in microbial diversity does not necessarily enhance soil functioning, improving soil properties can positively impact overall pasture health^6^. In addition, microbial diversity can serve as an indicator of soil biological quality due to its rapid response to modifications in pastures^35^. Therefore, soil fertility management should aim to improve soil properties in addition to increasing microbial diversity.

Effective fertility management can promote soil health and pasture productivity. Our findings suggest that the enrichment of Nitrospirae, Proteobacteria, Chloroflexi, Bacteroidetes, and *Candidatun* Rokubacteria can be associated to soil health and homeostasis through. These phyla would also be harboring species enriched with genes associated with aerobic ammonia oxidation, nitrate reduction, and aromatic compound degradation. Further research is needed to explore these interactions in greater depth and across a variety of pasture contexts, such as functional metagenomic analyses based on Shotgun sequencing and Metatranscriptomics. These advanced genomic approaches can provide a more comprehensive understanding of soil microbiome function.

## Methods

### Study site

According to the history provided by the owners, the areas have been subjected to intensive grazing, often exceeding the appropriate animal stocking rate. The management of these pastures was predominantly chemical, with weed control and occasional applications of nitrogen-based fertilizers. To maintain sample variability, two Mesoregions of the state of Pernambuco, Brazil—Agreste and Mata—were selected for the study (Fig. S1, Table S2). The climate of these zones was classified as tropical with dry summer (As), according to Köppen’s climate classification^37^. The herds in these regions were predominantly cattle fed under grazing a regime (*Brachiaria* sp.). Collections in each Mesoregion were made in three municipalities, where two pasture areas with differences in Normalized Difference Vegetation Index (NDVI) and N content of the aerial part of the plants were studied (Fig. S2), totaling twelve enclosures or experimental units. The selected areas featured soils with varying chemical properties, particularly base saturation (V%), covering an extensive territory with distances between sites ranging from a few meters (in adjacent enclosures) to approximately 200 km.

### Elaboration of NDVI maps

Maps of the regions under study were created using panchromatic and multispectral images from the CBERS-04A satellite’s WPM sensor (L4), which were obtained from the INPE website (http://www.dgi.inpe.br). The images generated by this sensor had multispectral and panchromatic resolutions of 8 and 2 m, respectively. QGIS version 3.16.10 software was used to process these images, with the SIRGAS 2000/UTM zones 24S and 25S (EPSG:4674) coordinate system. RGB bands were merged to evaluate vegetation cover and determine suitable areas for collection. Atmospheric correction was then applied using the Semi-Automatic Classification Plugin version 7.0.0.1^38^, and the red (R, band 3: 0.63 – 0.69 µm) and near-infrared (NIR, band 4: 0.77–0.89 µm) spectral bands were used to calculate NDVI = (NIR − R)/(NIR + R). In QGIS, the Pansharpening function was used to merge the high-resolution panchromatic band with the NDVI color composition.

### Collection procedures

In each of the twelve enclosures, four quadrants with an area of 1 ha were delimited, used as a reference for estimates of the average NDVI, an effective index for inferring the productive status of pastures^39^ and therefore the productive potential of the soil. In each municipality, two pastures were selected for the study, cultivated within a radius of up to 10 km. These pastures were previously classified based on the average NDVI and base saturation as less fertile (V% < 50%) and more fertile (V% ≥ 50%), with the aim of obtaining two contrasting scenarios from the same sample area. In each quadrant, 10 random subsamples were collected from the 0–20 cm soil layer to form a composite sample, resulting in 48 independent samples (experimental plots). About 1 kg of soil was incorporated for each plot and stored in plastic bags closed with elastic bands, keeping a paper towel at the opening to allow gas exchange. The aerial part of the pastures was also harvested for analysis of total N content, cutting the plant portion above 10 cm from the surface next to each soil collection (subsamples). Part of the soil was spread on absorbent paper on a bench and dried at room temperature, sieved through a 2 mm mesh, and used for chemical analysis to determine fertility. Other soil fractions were stored in 2 mL plastic microtubes and stored in a freezer at – 80 °C to preserve genomic DNA.

### Chemical analyses of soil and plant material

The chemical analyses to determine soil fertility were carried out according to the protocols in the EMBRAPA soil analysis manual^40^, testing soil pH in water (1:2.5 v:v) and in CaCl_2_ (1:2.5 v:v), as well as the main soluble macronutrients (P, K^+^, Ca^2+^, and Mg^2+^), calculations of cation exchange capacity (CEC), base saturation (V%), and levels of Na^+^, Al^3+^, and H + Al (potential acidity).

Total organic carbon (TOC) was measured using the method described by Yeomans and Bremner^41^, which is based on the reduction of dichromate (Cr_2_O_7_^2−^) by organic carbon compounds. In a digestion tube, 0.1 g of each soil sample was weighed. Then 5 mL of 0.167 mol L^−1^ K_2_Cr_2_O_7_ and 10 mL of concentrated H_2_SO_4_ were added. The tubes were placed in a digester block and kept at 170 °C for 30 min. After digestion, the samples at room temperature were transferred to Erlenmeyer flasks and added with 5 mL of H_3_PO_4_ to allow clear visualization of the titration turning point. Three drops of 1% diphenylamine indicator were then added and the remaining ${{{\text{Cr}}}_{2}{\text{O}}}_{7}^{2-}$ was determined by titration of the excess Cr^3+^ with 0.4 mol L^−1^ ammoniacal ferrous sulfate [(NH_4_)_2_Fe(SO_4_)_2_·6H_2_O]. The TOC contents were calculated according to the recommendations and mathematical equation described by Cantarella et al.^42^.

Leaf nitrogen was measured using an adapted sulfur digestion method^43^. The digest solution was prepared by adding substances in sequence to a 1000 mL beaker: 175 mL H_2_O, 3.6 g Na_2_SeO_3_, 21.39 g Na_2_SO_4_, 4.0 g CuSO_4_ 5H_2_O, and finally 200 mL of H_2_SO_4_. Plant samples (100 mg) were ground, sieved (2 mm), and digested with 7 mL of solution. The digester block temperature was raised by 50 °C every 30 min until reaching 350 °C and held until the solution became colorless or slightly greenish. Digestion tubes were attached to a nitrogen distiller and slowly filled with 18 mol L^−1^ NaOH until turning greenish brown. A conical flask with 10 mL of boric acid indicator solution [20 g H_3_BO_3_; 1000 mL H_2_O; 15 mL of a 0.1% alcoholic solution of C_21_H_14_Br_4_O_5_S; and 6 mL of a 0.1% alcoholic solution of C_15_H_15_N_3_O_2_] was placed at the distiller outlet continued until the volume doubled and turned slightly greenish. After digestion, the solution was titrated with 0.02 mol L^−1^ H_2_SO_4_ until the indicator turned from green to blue. The volume (V) used was recorded in mL and nitrogen percentage (%N) was calculated using %N = 0.28 V^43^.

### Genomic DNA extraction from soil and preparation of 16S rRNA libraries

Genomic DNA was extracted from a small sample of soil (0.4 g) using the DNeasy® PowerSoil® Kit (QIAGEN Inc., Valencia, CA, USA). Following the manufacturer’s instructions, the concentration and quality of the purified DNA were evaluated using a NanoDrop® 2000 spectrophotometer from Thermo Fisher Scientific Inc. (Waltham, MA, USA). Next, the three highest-quality repeats from four quadrants were selected to prepare our amplicon libraries, resulting in a total of 36 samples.

Sequencing libraries were constructed by amplifying the V3-V4 variable region of the 16S rRNA gene using Bakt_341F (5′-CCT ACG GGN GGC WGC AG-3′) and Bakt_805R (5′-GAC TAC HVG GGT ATC TAA TCC-3′) primers^44^. In conjunction with primers, the 16S rRNA gene amplicon sequencing library was generated using Herculase II Fusion DNA Polymerase (© Agilent Technologies, Inc., Santa Clara, CA, USA) and the Nextera XT v2 Index Kit (© Illumina, Inc., San Diego, CA, USA), following the manufacturer’s guidelines at Macrogen in Seul, South Korea. Sequencing was performed on an Illumina® MiSeq® using a v3 flow cell. A library concentration of 3 pM was loaded, with a 30% spike-in of the Illumina® PhiX control DNA library, following the manufacturer’s guidelines. The binary base call (BCL) files, which are the raw data files generated by Illumina sequencers, were converted into sequence data in FASTQ format using the bcl2fastq v2.20 software (© Illumina). The sequences were then demultiplexed and the barcodes were removed.

### Processing of raw genetic data

A total of 2,674,738 raw sequence pairs (forward and reverse) obtained through Illumina MiSeq sequencing were analyzed using the ‘DADA2’ pipeline version 1.16^45^ in R version v.4.2.3^46^ in conjunction with RStudio 2023.03.0 Build 386^47^. The FIGARO tools^48^ were utilized to optimize the truncation length parameters using the “filterAndTrim” R function (290 bases for forward reads and 260 bases for reverse reads). According to this tool, forward and reverse reads with more than 2 and 5 expected errors (maxEE), respectively, were discarded. Next, reads were truncated at the first instance of a quality score (truncQ) less than or equal to two. Error rates of the sequences were calculated using the “learnErrors” function, a machine learning-based algorithm. Amplicon sequence variants (ASVs) were inferred using the “given” function, and paired reads were merged by applying the outputs of the previous functions to the input of “mergePairs”. Chimeric sequences were identified using the “removeBimeraDenovo” function and taxonomic assignments were given to the remaining sequences based on the Silva SSU 138 (modified) database^49^, using the “IdTaxa” algorithm from the ‘DECIPHER’ v 2.20 R library^50^, which is considered a method with better classification performance than the standard set by the naive Bayesian classifier^51^. The data processing resulted in 854,980 high-quality sequences, allowing for the identification of 13,470 Amplicon Sequence Variants (ASV) when combining the information from the 36 composite soil samples. The paired and chimera-free sequences, along with their respective BioSample assignments, were deposited in the NCBI repository under project code PRJNA753707 (https://www.ncbi.nlm.nih.gov/).

### Statistical analysis

All data analyses were computed using resources developed for R language v.4.2.3^46^. The soils of the six municipalities were classified with K-Means to identify groups with contrasting fertility levels, using the Hartigan-Wong algorithm (R ‘stats’) adjusted to return a cluster center for each input point. Principal component analysis was performed (R ‘vegan’) to assist in the choice of clusters, allowing the identification of the contribution of soil chemical attributes and foliar nitrogen on the main dimensions of the multivariate model. The most characteristic variables of each dimension were pointed out by factor analysis (R ‘FactoMineR’), according to the method published by Husson et al.^52^.

After defining the soil property clusters by K-means, soils from the intermediate cluster were not considered as they might introduce undesirable noise for the purpose of this study: to contrast the impact of two extremes on bacterial communities. After the construction of the HF soil (high fertility—15 samples) and LF soil (low fertility—10 samples) clusters, the chemical attributes were compared by the t-test and Wilcoxon signed-rank test (R ‘agricolae’), comparing the means and contrasts between the dispersions of the pairs, respectively, both at a 5% similarity level. All probabilities were adjusted using the Benjamini and Hochberg method, a powerful technique that controls the False Discovery Rate (FDR). Variables expressed as percentages (y%) were transformed using the function sin^−1^ [√(y%⁄100)]180/π. These transformations are recommended for controlling error rates in biological data, resulting in acceptable residual analysis versus fit plots and producing p-values like the original data^53^.

The study focused on abundant bacterial communities, retaining ASVs with a relative abundance greater than 0.01%. Canonical correlation analysis (CCA) was used to identify and measure associations between the set of genetics and the environmental variables (Fig. 3), testing the significance rate of chemical attributes through the Mantel test, with Pearson correlations (R ‘vegan’). The same R library was used to estimate Alpha-Diversity metrics. Among these, the Shannon and Simpson indices were converted into effective or equivalent species numbers, also known as Hill numbers, which considered the number of equally abundant species necessary to produce the observed diversity value. To calculate differential abundance between atomic ranks, the Random Forest algorithm with the Kruskal–Wallis rank sum differential test was used, based on White et al.^54^. In this case, the differences in the mean importance of each taxon in the decision tree (MDG—Mean Decrease Gini) were calculated, a forest-wide weighted average of the decrease in the Gini impurity metric between daughter and parent nodes that a taxon is splitting.

Microbial co-occurrence patterns were analyzed using the SparCC association measure through the SPIEC-EASI approach (SParse Inverse Covariance Estimation for Ecological Association Inference) of the R SpiceEasi package^55^. The data were normalized based on the method of the R NetCoMi package; a technique suitable for identifying groups of highly correlated species^56^. To do so, the ten closest samples (Bray–Curtis dissimilarity) within each of the two clusters were selected, and ASVs with a relative frequency lower than 0.1% were discarded. The network graphs were constructed using Gephi software v. 0.10.1^57^, where disconnected nodes and edges with weights lower than 0.6 or p-value greater than 0.01 were hidden. In these approaches, the nodes (ASVs) were classified into modules to analyze the connectivity of sub-communities that made up the network. Module eigengene analyses were also performed, which is the association of the first principal component of each detected module with environmental factors. In these analyses, all probabilities were also adjusted by the Benjamini and Hochberg method.

The functional prediction analysis was based on the association of 16S rRNA sequences with the collection of prokaryotic functional profiles deposited in the FAProTax database^58^. The predicted processes were subjected to correlation and differential abundance tests by the effect size method of linear discriminant analysis (LEfSe) and DMG measure through the R ‘microeco’ library, based on Segata et al.^59^.

To validate the results of the 16S-based metagenomic prediction, three random genomic DNA samples were submitted to Shotgun metagenomic sequencing using the Illumina NovaSeq PE (150 bp), following the manufacturer’s guidelines at Novogene Inc. in Sacramento, CA, USA. After the Shotgun sequencing was performed, the FASTQ reads were filtered according to quality score using Trimmomatic (v. 0.39), ensuring high-quality sequences. Subsequently, the high-quality reads were assembled into contigs using the MEGAHIT tool (v. 1.2.9), a single-node assembler for NGS reads. Following assembly, protein sequences were classified using the Prodigal tool (v. 2.60). The predicted genes were then submitted for functional annotation using the eggNOG-mapper (v. 2.1.12, http://eggnog-mapper.embl.de/). This step facilitated comprehension of the functional capabilities of the soil microbial communities. Subsequently, various KEGG Orthology (https://www.genome.jp) pathways (KO) were examined, transformed into relative frequencies, and correlated with the corresponding functional processes predicted from the 16S gene via amplicon sequencing (Shotgun vs FAProTax). The sole use of Shotgun sequencing data in this work was not for in-depth functional studies, as they were beyond the primary objective of the study.

All heatmaps used in the composition of Figs. 2, 3, 5, and 6 were constructed with the ‘heatmaply’ library (v. 1.5.0). In this case, the correlations and hypothesis tests between variables of two data matrices (rows and columns of the heatmaps) were computed using the “cor.test” function from the ‘stats’ library (v. 4.3.1). All other graphs were constructed using resources provided by the ‘ggplot2’ package^60^. The composition of the images within the figures was accomplished using the “grid.arrange” and “arrangeGrob” functions from the ‘gridExtra’ library (v. 2.3).

### Supplementary Information

Supplementary Information.

## Data Availability

The genetic sequences and corresponding BioSample assignments from this study can be accessed in the NCBI repository, under project code PRJNA753707. Additional data may be made available upon prior request to the first author (corresponding author).

## Abbreviations

ASV: Amplicon sequence variant
CCA: Canonical correlation analysis
CEC: Cation exchange capacity
FAProTax: Functional annotation of prokaryotic taxa
HF: Soils with higher fertility grouped by K-means clustering
K-means: Clustering algorithm by unsupervised learning method
LDA: Linear discriminant analysis
LF: Soils with low fertility grouped by K-means clustering
MDG: Mean decrease Gini in the random forest model
NDVI: Normalized difference vegetation index
PCA: Principal component analysis
RF: Random Forest algorithm
SparCC: Sparse correlations for compositional data
SS: Sum of squares
TOC: Total organic carbon
V%: Percentage of base saturation in the soil
