# Supplementary material for: Soil fertility impact on recruitment and diversity of the soil microbiome in sub-humid tropical pastures in Northeastern Brazil
Source: Sci Rep. 2024 Feb 16;14:3919. doi: 10.1038/s41598-024-54221-7 (PMC10873301; doi:10.1038/s41598-024-54221-7)
Supplement: Supplementary file 1 — Supplementary Information. [file 41598_2024_54221_MOESM1_ESM.pdf]

SUPPLEMENTARY MATERIAL

(Scientific Reports)

Soil fertility impact on recruitment and diversity of the soil microbiome in sub-humid tropical pastures in Northeastern Brazil

Diogo Paes da Costa [1,*], Thallyta das Graças Espíndola da Silva [1], Ademir Sérgio Ferreira Araujo [2], Arthur Prudêncio de Araujo Pereira [3], Lucas William Mendes [4], Wisraiane dos Santos Borges [1], Rafaela Felix da França [1], Carlos Alberto Fragoso de Souza [1], Bruno Alves da Silva [1], Renata Oliveira Silva [1], Erika Valente de Medeiros [1]

[1] Microbiology and Enzimology Lab., Federal University of Agreste Pernambuco, Garanhuns, PE 55292-270, Brazil.
[2] Soil Quality Lab., Agricultural Science Center, Federal University of Piauí, Teresina, PI 64049-550, Brazil.
[3] Microbial Ecology and Biotechnology Lab., Federal University of Ceará, Fortaleza, CE 60020-181, Brazil.
[4] Center for Nuclear Energy in Agriculture, University of Sao Paulo, Piracicaba, SP 13400-970, Brazil.
*Email: diogopaes1@gmail.com

DOI: https://doi.org/10.1038/s41598-024-54221-7

SUPPLEMENTARY TABLES

Table S1. Statistical summary of the chemical attributes of HF and LF clusters.

Measure	HF		LF		p-value [a]	
	mean	sd [b]	mean	sd	t-test	Wilcoxon
pH (H_2O)	6.4	0.3	5.8	5.8	**0.00**	**0.00**
pH ($CaCl_2$)	5.7	0.5	4.7	4.7	**0.00**	**0.00**
Ca^{2+} (cmolc dm^{-3})	4.5	2.0	1.2	1.2	**0.00**	**0.00**
Mg^{2+} (cmolc dm^{-3})	3.8	1.7	1.2	1.2	**0.00**	**0.00**
P (mg dm^{-3})	13.0	10.7	19.8	19.8	0.10	0.06
Na^{+} (cmolc dm^{-3})	0.2	0.1	0.1	0.1	0.16	0.08
K^{+} (cmolc dm^{-3})	0.4	0.2	0.2	0.2	0.10	0.19
H+Al (cmolc dm^{-3})	4.0	1.3	4.8	4.8	0.08	0.21
Al^{3+} (cmolc dm^{-3})	0.1	0.1	0.2	0.2	**0.01**	**0.00**
TOC (g kg^{-1}) [c]	8.9	3.7	3.9	3.9	**0.00**	**0.00**
CEC (cmolc dm^{-3}) [d]	12.7	3.8	7.5	7.5	**0.00**	**0.00**
V%	68.1	8.1	31.4	31.4	**0.00**	**0.00**

[a] P-values highlighted in bold were considered significant ($p < 0.05$) according to statistical analysis.
[b] Standard deviation.
[c] Total organic carbon; [d] Cation exchange capacity.

Table S2. Localization and climatic characteristics of the local sampled in the Agreste Meridional and Zona da Mata Mesoregions, Pernambuco state, Brazil.

**n**	**Region** [a]	**Site**	**Temperature (°C)**			**Pre.** [b]	**Alt.** [c]	**Coordinates** [d]	
			mean	min	max	mm	m	latitude	longitude
1	Agreste	Brejão	21	16.6	25.4	1051	822	-8.99247	-36.53902
2		Brejão					813	-8.99400	-36.53689
3		Garanhuns	20.4	16.1	24.8	873	728	-8.97391	-36.45463
4		Garanhuns					725	-8.97491	-36.45547
5		São João	21.1	16.7	25.6	885	680	-8.81075	-36.40785
6		São João					689	-8.80925	-36.40841
7	Mata	Carpina	24	19.4	28.7	1082	158	-7.86081	-35.21356
8		Carpina					132	-7.86356	-35.21304
9		Paudalho	24.6	20.4	28.9	1239	129	-7.85693	-35.28175
10		Paudalho					152	-7.85728	-35.28315
11		Vicência	24.9	19.9	29.9	1168	124	-7.63461	-35.30874
12		Vicência					100	-7.63432	-35.30498

[a] Köppen-Geiger climate classification: tropical climate with dry summer (As).
[b] Average annual precipitation.
[c] Average altitude relative to sea level.
[d] Geographic coordinates (Datum WGS84).

Table S3. Correlation analysis between chemical attributes and the two main axes of PCA.

Variable	Dimension 1	p-value [(a)]	Dimension 2	p-value [(a)]
Al^{+3}	-0.45	**0.024**	0.05	0.798
Ca^{+}	0.94	**0.000**	0.13	0.529
CEC [(b)]	0.87	**0.000**	-0.36	0.075
H+Al	-0.27	0.186	-0.70	**0.000**
K^{+}	0.44	**0.028**	0.73	**0.000**
Mg^{2+}	0.80	**0.000**	-0.55	**0.004**
Na^{+}	0.41	**0.040**	0.65	**0.000**
N_{grass}	0.32	0.118	0.45	**0.025**
P	-0.09	0.653	0.70	**0.000**
pH_{CaCl2}	0.91	**0.000**	-0.02	0.913
pH_{H2O}	0.93	**0.000**	0.06	0.771
TOC [(c)]	0.76	**0.000**	-0.41	**0.043**
V%	0.90	**0.000**	0.08	0.706

[(a)] P-values highlighted in bold were considered significant ($p < 0.05$) according to factorial analysis.
[(b)] Cation exchange capacity.
[(c)] Total organic carbon.

Table S4. Statistical summary of microbial alpha-diversity indices of HF and HL pastures.

**Measure**	**HF**		**LF**		**Wilcoxon test**
	mean	sd [a]	mean	sd [a]	p-value [b]
Observed [c]	910.9	120.8	781.6	104.1	1.58E-02
Shannon	6.392	0.130	6.185	0.150	1.47E-03
Simpson	0.997	0.001	0.997	0.001	1.86E-03
Shannon (Effective)	601.9	77.7	490.2	70.3	1.47E-03
Simpson (Effective)	408.0	73.3	300.9	66.2	1.86E-03

[a] Standard deviation.

[b] P-values < 0.05 indicated significant differences between HF and LF soils.

[c] Species richness was represented through the count of unique ASVs. Effective: Shannon and Simpson values converted into effective or equivalent species numbers (also known as Hill numbers).

Table S5. Main metrics of the microbial co-occurrence network in pasture clusters.

Parameters / environments [(a)]	HF	LF
Number of vertex [(b)]	138	173
Number of edges [(c)]	638	4026
Positive edges (%) [(d)]	66	58
M1 module (%) [(e)]	38.41	48.55
Network diameter [(f)]	9.00	4.00
Average degree [(g)]	9.25	46.54
Average path length [(h)]	2.76	1.70
Centrality [(i)]	0.20	0.62
Clustering coefficient [(j)]	0.89	0.87
Density [(k)]	0.07	0.27
Heterogeneity [(l)]	1.30	0.83

[(a)] Soil fertility: HF - high fertility and LF - low fertility.
[(b)] Node: each object (ASV) in a network.
[(c)] Edge (link): a link between each pair of nodes.
[(d)] Positive edge: SparCC > 0 and p-value < 0.05.
[(e)] Network modules represent important ecological units, which can have significant implications for biological or ecological functions [1].
[(f)] Network diameter is the longer of the pairwise shortest path lengths or the size of the largest connected component, being quantified by the average number of edges. The diameter may or may not be correlated with the edge number [2, 3].
[(g)] Average degree or node connectivity is number of your direct connections to other nodes [2].
[(h)] Average path length or average shortest path length is an indicator of system performance or the degree of compaction of the microbial structure. In other words, it indicates the average number of steps required to get from one node to another in the network [4].
[(i)] Betweenness centrality, average importance of nodes in the network, and all modes, being higher in nodes connected to more influential (more connected) neighbors. Therefore, it measures the importance of nodes by their frequency of occurrence on paths connecting other nodes [3, 5].
[(j)] The degree to which nodes in a graph tend to cluster, defined as the average proportion of connections between neighbors that are made and the number of all possible connections. In other words, it quantifies the tendency of the graph to be divided into subunits. Values >0.4 suggest that the network has a modular structure [6]. High modularity signifies densely connected nodes within certain groups and sparse inter-group connections in the network [5].
[(k)] Measure of the integrity and effectiveness of the network, it is the observed fraction of real connections by possible collections. Density represents the ratio of observed microbial associations to all potential associations, given the network's nodes [3].
[(l)] Proportional to the modularity of the habitat, it quantifies the number of distinct modular structures [7].

Table S6. Relative composition (%) of phyla in the main modules of co-occurrence networks.

	HF (M1)	HF (M2)	LF (M1)	LF (M2)
Actinobacteria	56.9	66.7	53.0	41.8
Proteobacteria	13.7	20.0	26.5	12.7
Acidobacteria	5.9	0.0	9.6	16.5
Firmicutes	11.8	6.7	6.0	24.0
Verrucomicrobia	3.9	0.0	2.4	3.8
Choroflexi	7.8	6.7	1.2	0.0
Planctomycetes	0.0	0.0	1.2	1.3

[(a)] Soil fertility clusters: HF - high fertility and LF - low fertility. M1 and M2 are the primary and secondary modules of microbial networks.

SUPPLEMENTARY FIGURES

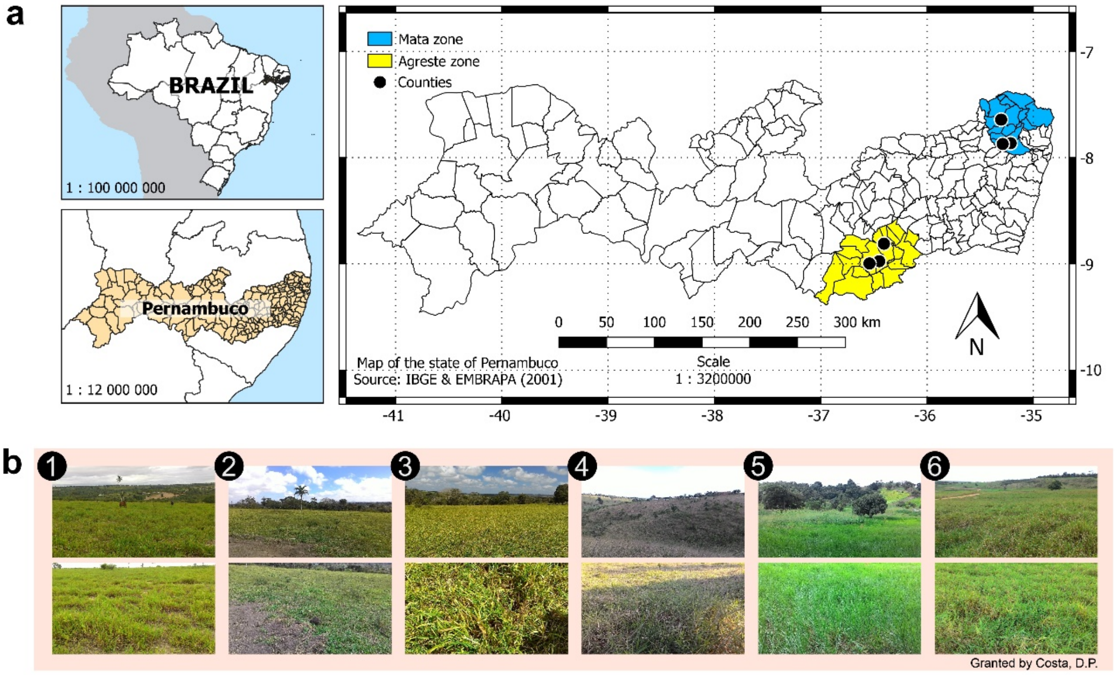

Figure S1. Identification of collection areas in the state of Pernambuco, Northeast of Brazil. (a) Map of the state showing the Microregions (colors) and the corresponding sampled municipalities (black circles). In total, 12 pasture areas were studied, forming four composite soil samples from each, totaling 48 samples. **(b)** Photograph showing areas and details of pastures in the six visited municipalities: 1 - São João, 2 - Brejão, and 3 - Garanhuns (Agreste Region); 4 - Carpina, 5 - Paudalho, and 6 - Vicência (Zona da Mata Region).

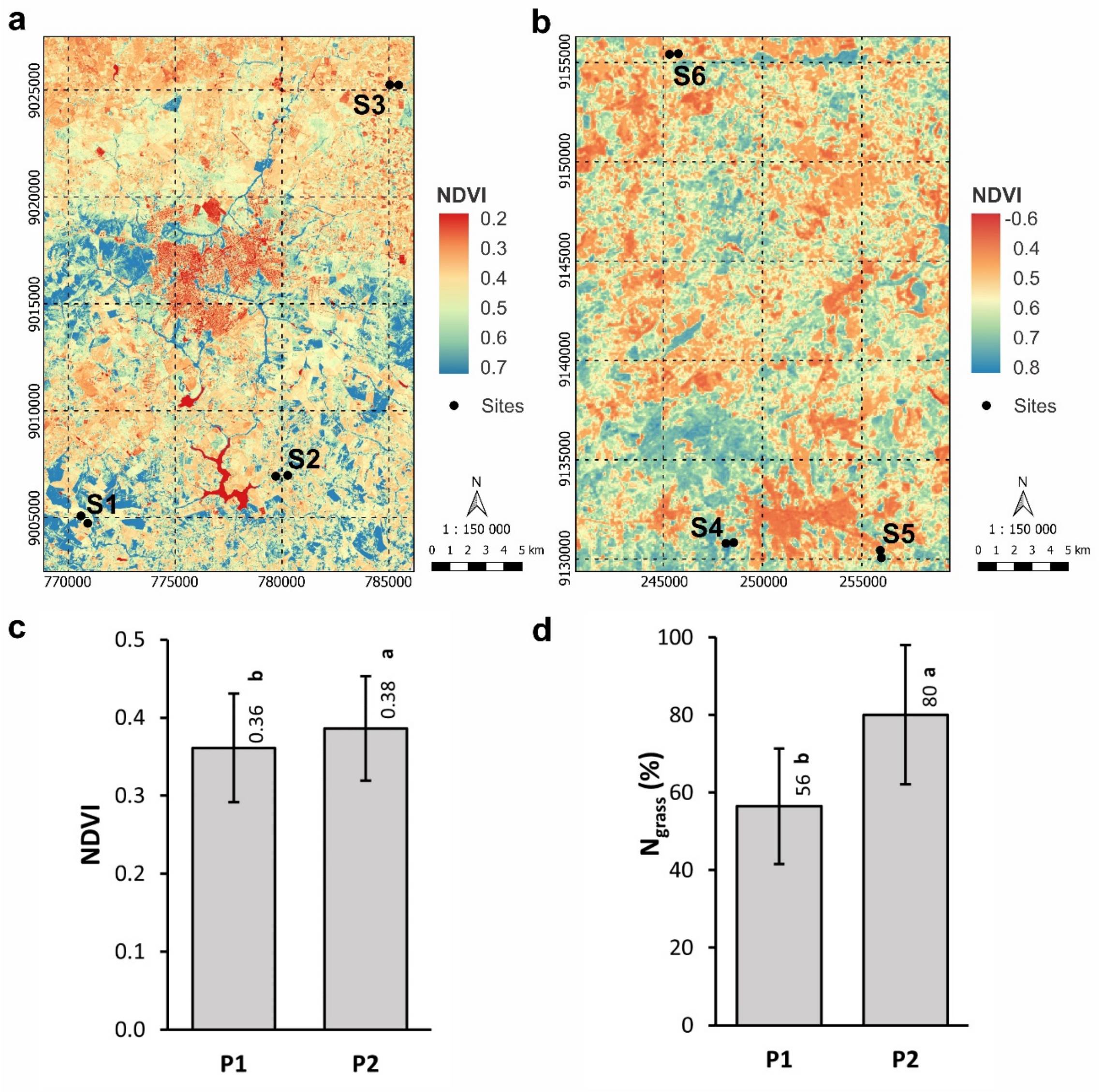

Figure S2. Spatial location and vegetation status of the sample sites. The coordinates of the six sites located in the Agreste **(a)** and Mata **(b)** zones were plotted on maps of the Normalized Difference Vegetation Index (NDVI). Each site was composed of two pastures that differed in NDVI **(c)** and nitrogen concentration in the aerial part of the pastures **(d)**, according to the comparison between means by the t-test ($p < 0.05$), with these values being associated with soil chemical attributes and climatic conditions of each location. For comparative purposes, the pastures were named P1 (less productive) and P2 (more productive), each one being formed by pastures from all sites.

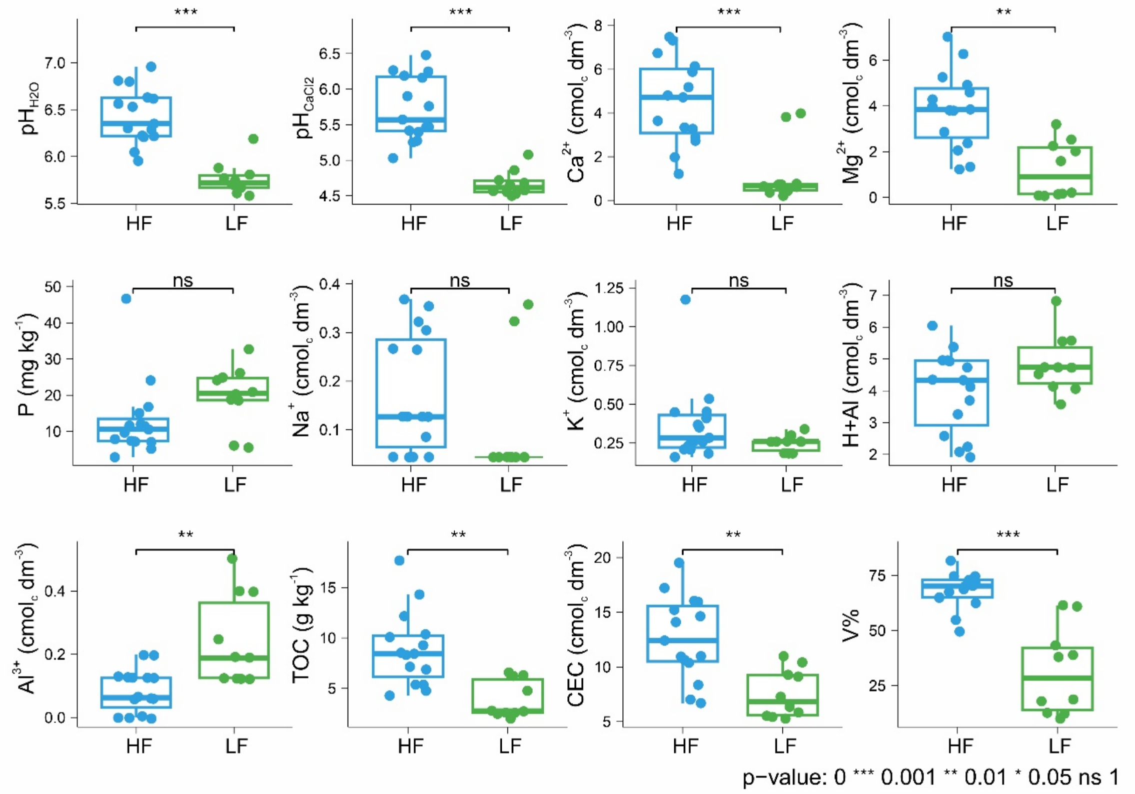

Figure S3. Dispersion of edaphic variables in pastures with high (HF) and low (LF) fertility levels. Comparisons with more than one asterisk (*) indicate that HF and LF clusters had distinct means and were representative of populations with distinct distributions, according to t-tests (parametric) and Wilcoxon signed-rank tests (non-parametric), respectively, both at the 5% significance level.

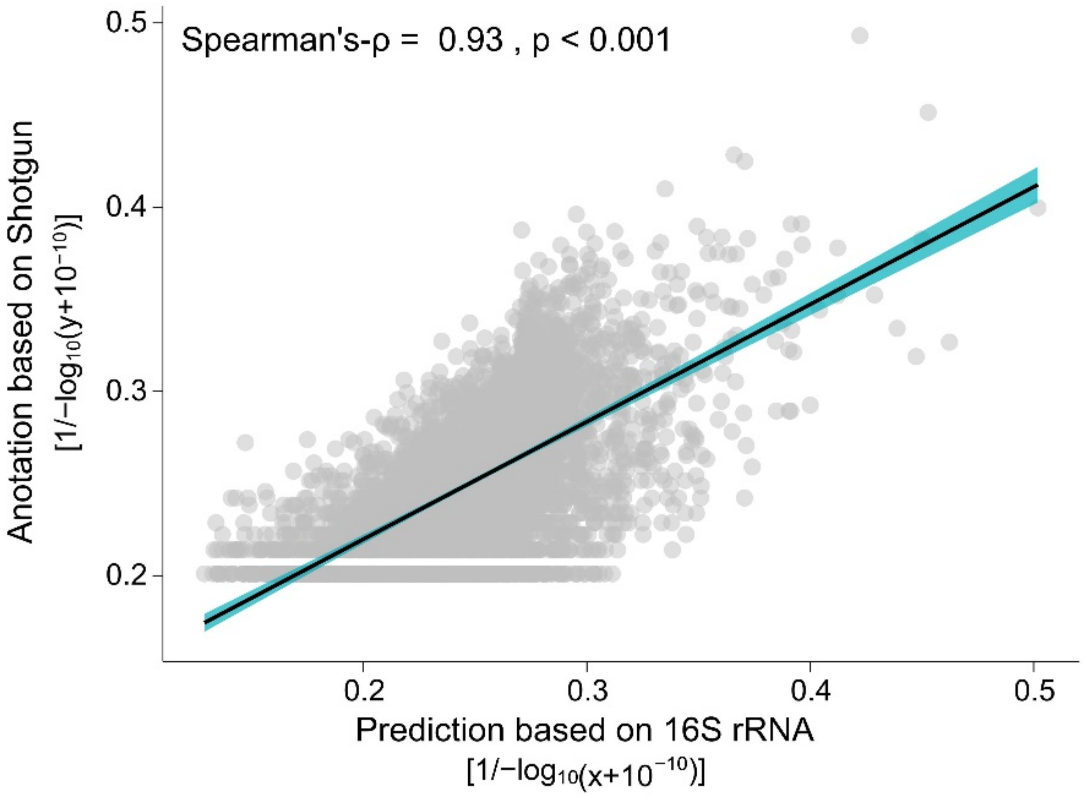

Figure S4. Associations between metagenomic prediction based on the 16S rRNA gene and annotation based on prokaryotic origin contigs obtained from Shotgun metagenomic sequencing were examined. The relative frequency data were incremented by a small value (10^{-10}) to eliminate null counts and then transformed by the expression $1/-\log_{10}(x)$. The Spearman correlation coefficient was used to test the degree of correlation between the data.

References

[1] Zhang, G. *et al*. Modules in robust but low-efficiency phyllosphere fungal networks drive saponin accumulation in leaves of different Panax species. *Environ. Microbiome*, **18**, 57. https://doi.org/10.1186/s40793-023-00516-7 (2023).

[2] Barberán, A. *et al*. Using network analysis to explore co-occurrence patterns in soil microbial communities. *ISME J.* **6**, 343–351 https://doi.org/10.1038/ismej.2011.119 (2012).

[3] Ma, B. *et al*. Earth microbial co-occurrence network reveals interconnection pattern across microbiomes. *Microbiome*. **8**, 82 (2020). https://doi.org/10.1186/s40168-020-00857-2

[4] Chen, F. *et al*. The average path length of scale free networks. *Commun. Nonlinear Sci*. **13**, 1405–1410. https://doi.org/10.1016/j.cnsns.2006.12.003 (2008).

[5] Layeghifard, M., Hwang, D.M. & Guttman, D.S. Disentangling Interactions in the Microbiome: A Network Perspective. *Trends Microbiol*. **25**, 217-228. https://doi.org/10.1016/j.tim.2016.11.008 (2017).

[6] Newman, M.E.J. Modularity and community structure in networks. *Proc. Natl. Acad. Sci. USA*. **103**, 8577. https://doi.org/10.1073/pnas.0601602103 (2006).

[7] Jacob, R., Harikrishnan, K.P., Misra, R., Ambika, G. Measure for degree heterogeneity in complex networks and its application to recurrence network analysis. *R. Soc. Open Sci*. 4, 160757. https://doi.org/10.1098/rsos.160757 (2017).
